# Spreading the Joy of Literacy: A community-based survey of parents by parents

**DOI:** 10.1371/journal.pone.0361893

**Published:** 2026-11-03

**Authors:** Sarah Collins-McGowan, Claude Andrew Aligne, Kelly McDermott, Candace Cabral, Ana Pilar Ramos Benitez, Melissa Lomack, Nicole Mayo

**Affiliations:** Department of Pediatrics, General Pediatric Division, Hoekelman Center, University of Rochester School of Medicine and Dentistry, Rochester, New York, United States of America; Lamar University, UNITED STATES OF AMERICA

## Abstract

**Background:**

Literacy is an important social determinant of health. Failure to develop emergent literacy skills before kindergarten is a risk factor for academic failure, poverty, incarceration, and early death. Reading comprehension depends on both language comprehension and alphabetic decoding skills. The Home Literacy Environment literature tends to focus on reading aloud to develop oral language but less is known about letter-themed tools.

**Design:**

Our hypothesis was that letter-themed toys and books would be acceptable to all families but that lower socio-economic status (SES) households would be less likely to have them. Using methods of Community Based Participatory Research, 3 caregivers with young children living in the City of Rochester, NY, completed IRB training and provided cultural context for decisions throughout this study. We surveyed a convenience sample of 156 caregivers at 10 community events. We showed a poster with pictures of alphabet-themed toys and asked caregivers to count how many they had in the home. ZIP code was used as a proxy for SES.

**Results:**

We surveyed 156 caregivers, excluding 13 responses because of missing information. Of the 143 remaining, 139 (97.2%) thought it was important that a child know most of the alphabet letters before kindergarten. 1 in 4 of our respondents didn’t have a single literacy toy in their home and an additional 1 in 6 had only one. We calculated a p value of 0.0004 for the hypothesis that there is a difference by SES in the number of these items in the home.

**Conclusions:**

Our results are consistent with the hypothesis that joyful letter-themed tools are acceptable to parents in general but that low-SES households are less likely to have them. Closing the gap in early alphabet exposure could contribute to reducing inequities in school success and lifelong health.

## Introduction

### Background

Literacy is an important social determinant of health. Failure to develop emergent literacy skills before kindergarten is a risk factor for academic failure, poverty, incarceration, early death, and intergenerational illness [1,2]. Research has established that young children need frequent exposure to the alphabet in order to build the skills needed for decoding the correspondences between language and letters [3,4]. In the United States, there is a large equity gap in kindergarten readiness [5,6]. Programs intended to overcome this gap, such as universal pre-kindergarten, have had mixed results, particularly for children from low socioeconomic status (SES) backgrounds [7,8], who are especially vulnerable to early literacy challenges [9,10].

There is a large body of literature now referred to as the Science of Reading [11,12]. While this term has taken on controversial political and cultural connotations in the “Reading Wars,” we are using it here to refer to the decades of research from multiple fields, in particular the evidence from trials of instructional interventions [13,14]. One influential heuristic from this research is the Simple View of Reading: reading comprehension = language comprehension x decoding [15]. Children with strong language skills but weak decoding abilities will not be able to read proficiently. Conversely, children who can decode words but have limited vocabulary and language comprehension will also struggle. The “x” sign in the metaphorical equation of the Simple View is to emphasize that this is not either/or but rather both/and. For example, interventions like Marie Clay’s Reading Recovery that stress the language half of the equation are significantly more effective when combined with interventions focused on decoding skills [14].

This framework was developed to apply to the vast majority of children learning to read alphabetic languages. There are subgroups of children, such as deaf children, who have different learning needs and for whom this framework may not be clearly applicable. Nevertheless, research with deaf children also demonstrates the importance of early exposure to the alphabet [16–19].

The Home Literacy Environment (HLE) has been an important topic of study. However, the literature on the HLE has largely focused on the presence of print materials, such as books, as well as shared reading practices [20], which primarily support oral language comprehension. Similarly, in daycare and preschool environments, much of the research has focused on using books for reading aloud and play [21,22]. There has been comparatively little attention paid to the presence of activities or items that may support decoding skills, such as alphabet-themed toys and books, and little is known about whether children have equitable access to these resources across socioeconomic groups [23].

### Objectives

Our primary objective was to determine if there were differences by SES among parents regarding the availability and perceptions of play-promoting early-literacy tools in the homes of preschool children. Our hypothesis was that all families would want these items but that low-SES families would be much less likely to have them.

## Methods

### Participants

A convenience sample of 156 caregivers in the greater Rochester, NY area was surveyed from 17/01/2024–24/08/2024. These caregivers primarily consisted of parents and grandparents of young children. Participants were recruited by tabling at 10 community events that occurred both in the City of Rochester and in the surrounding suburbs.

### Materials

This study was developed as Community Based Participatory Research (CBPR). It builds on previous community advocacy projects in the area of early literacy, which led to the development of a parent advisory board consisting of 3 caregivers with young children living in the City of Rochester. These caregivers completed IRB training prior to the start of this study and provided important social and cultural context for decisions throughout the development of this study. We worked with this group to develop a brief, anonymous, one-page instrument that asks about literacy-related items and behaviors in the home (S1 Appendix).

On the table was a poster with pictures of various items, which included a dry erase workbook, a dry erase board, letter magnets, books about ABCs, Alpha-Bot toys, and foam bath letters (Fig 1). Caregivers were asked to count how many of these, or similar items, they had in the home. The items ranged in price from $4.00 to $24.99. The list of items displayed was developed in partnership with our parent advisors, preschool teachers, and early literacy experts in our area. Surveyors were available to answer questions if needed.

**Fig 1:**
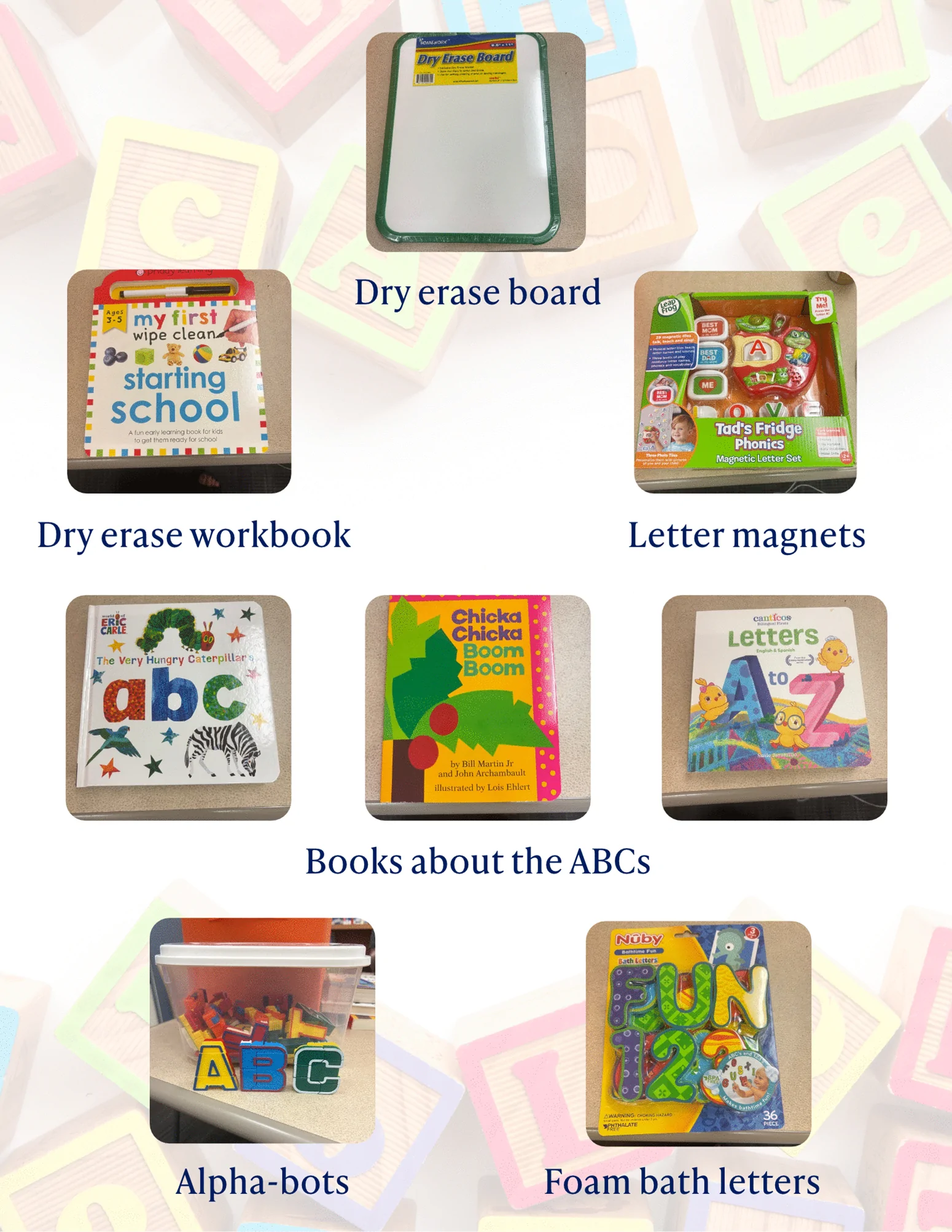
Poster of Alphabetic Books and Toys (figure is similar but not identical to the original image and is therefore used for illustrative purposes only).

Respondents were offered free alphabet toys at the tabling events. We noted that respondents in areas with higher average incomes often responded to the survey but did not take the toys.

ZIP code was used as a proxy for SES. Our parent advisers felt this was more culturally sensitive than asking about income. Rochester was historically subjected to red-lining practices, the impacts of which are still visible today in the economic segregation throughout the area. Median income data by zip code were pulled from the U.S. Census for 2024.

Before analyzing results, availability was dichotomized as having zero alphabetic items in the home versus having 5 + items in the home. Our group felt this was a meaningful difference based on our prior experience interacting with parents and teachers; we expected that people would tend to either have lots of these toys or none at all. We also looked at the distribution of incomes and grouped together ZIP codes from the City of Rochester and those from the suburbs in a dichotomized fashion using a median income cut-off of $55,000, which aligns with the 200% poverty threshold for families of 3–4 in 2024. We felt this was a more methodologically conservative approach to analyzing the data than looking at correlation across the spectrum and would be less likely to overestimate an association. The Fisher’s exact test was used to examine differences in number of items by income category (GraphPad QuickCalcs, 2024).

### Ethical considerations

The University of Rochester Research Subjects Review Board evaluated all aspects of this project, including the consent process, and determined that it met federal and university criteria for exemption (00008545). All potential participants received an information sheet in English or Spanish describing the purpose of the study, the voluntary nature of the participation, and the rights of the participants. Consent was obtained by the surveyor through verbal affirmation and completion of the survey.

## Results

We surveyed 156 caregivers in the greater Rochester, NY area. We excluded 13 responses for various reasons: their primary residence was out of state, they were grandparents who told us they were not sure about exactly what items were in the child’s home, or they didn’t answer questions such as their zip code or the number of items they own, leaving 143 total valid responses.

Among these, 139 (97.2%) thought it was important that a child know most of the letters of the alphabet before kindergarten, and 71 (49.7%) answered that the children in their life have a library card. We left an open-ended question at the end of the survey for “anything else you want to tell us” and typical comments were: “Early learning is very important,” “I think the exposure to these tools are very important for our children and their learning,” and “It is important for children to get the additional reading support to prevent later reading problems.”

There was wide variation in how many of the literacy items people had in the home (Figs 2 and 3), with a median of two items reported per household. 25% (n = 37) of respondents had 0 of the items, 55% (n = 78) had 1–4 items, and 20% (n = 28) had 5 or more items. When comparing zero items versus five or more, we found a significant difference between the two SES groups, with those in the higher SES group being more likely to have 5 + items at home than those in the lower SES group [p = 0.0004; Table 1]. Those living in ZIP code regions with a median income above $55,000 were significantly more likely to have more than two of these literacy items in their homes (Fisher’s exact p-value: 0.0002), which was reflected by a median of four items reported per household in that SES group.

**Table 1:** Item comparison by dichotomized income.

	5+ items at home	0 items at home	
High SES ZIP Code	16	5	21
Low SES Zip Code	12	32	44
Number of Respondents	28	37	65
	**Fisher's exact test**		
	The two-tailed P value equals 0.0004		

**Fig 2:**
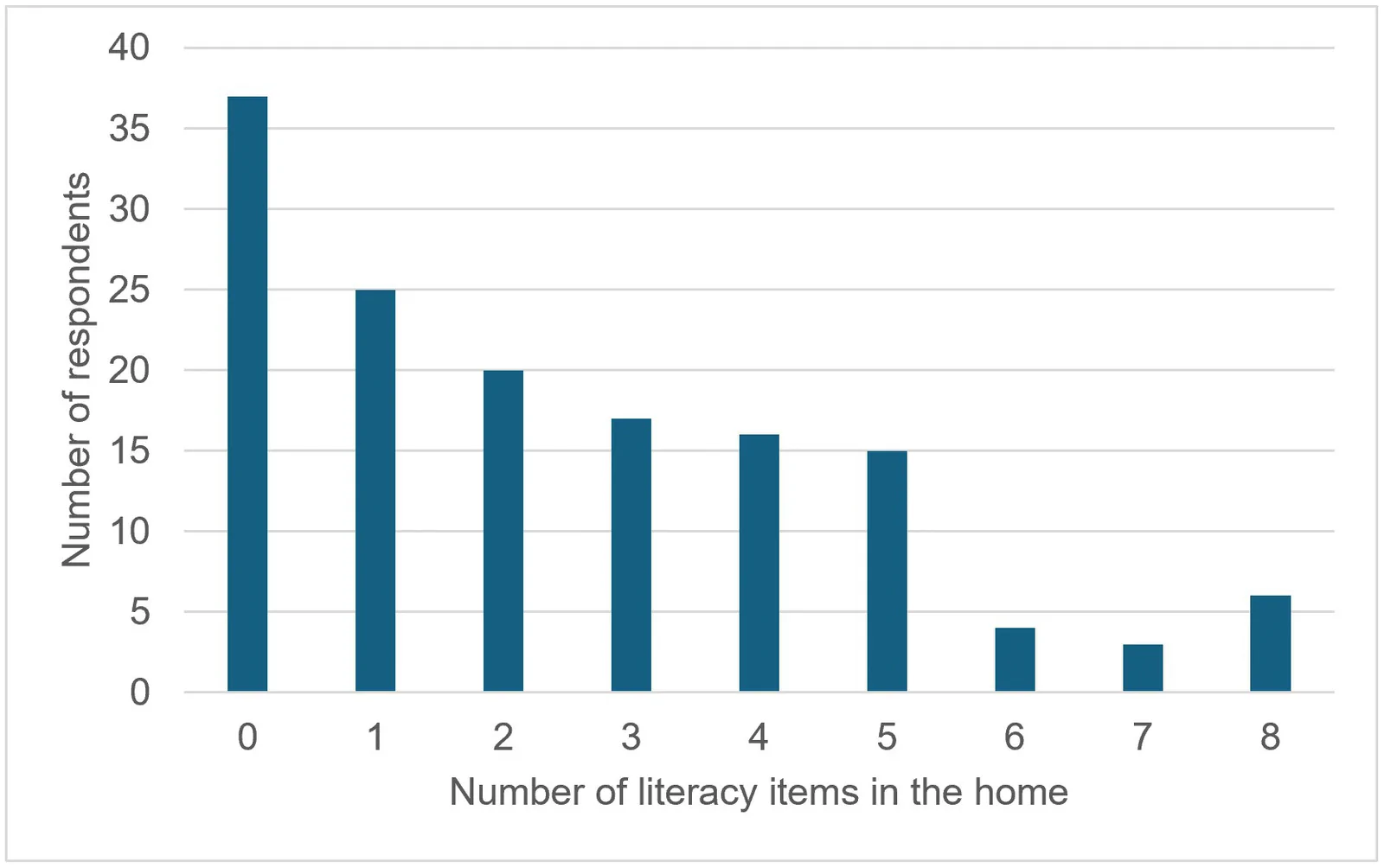
Distribution of literacy items.

**Fig 3:**
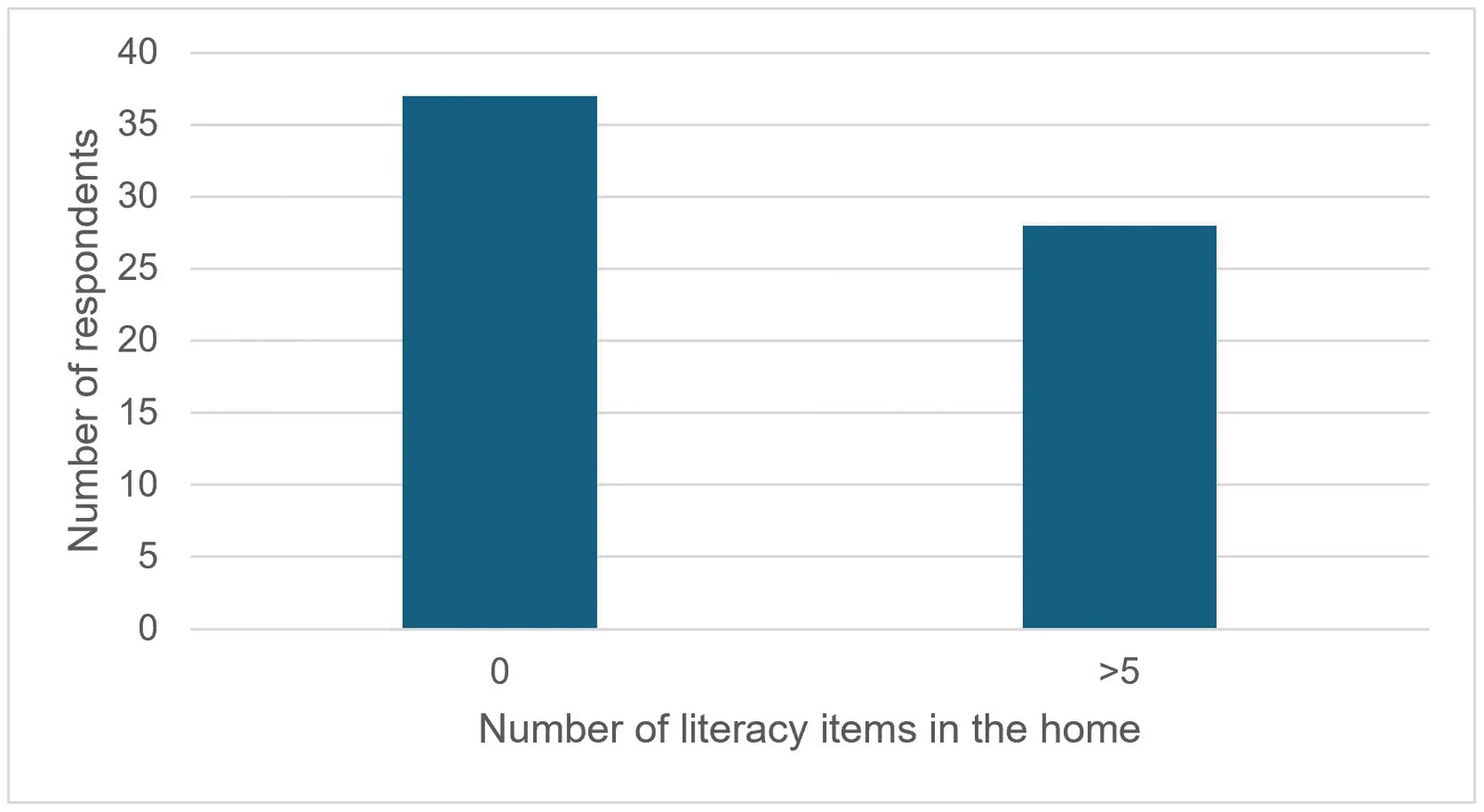
Dichotomized distribution of literacy items.

## Discussion

The results of this study are consistent with our hypothesis that alphabet toys for promoting emergent literacy skills are acceptable to parents in general but that low-SES households are less likely to have them available in the home. In our sample, 25% of respondents had zero alphabet items in their home.

Literacy begins at birth [24]. Learning to read is a civil right and a critical social determinant of health; increasing early literacy has the downstream effect of improving health and longevity as well as overcoming cyclical poverty [1,2]. The literature on precursor skills for literacy development is clear: children who can identify letter names, understand that letters represent sounds, and write their first names before kindergarten are more likely to be reading at grade level by first grade [1,2].

Numerous programs already exist to provide books to families and to encourage reading aloud to children [24,25]. With respect to the Simple View of Reading [15], this puts the emphasis on increasing oral language comprehension as opposed to increasing alphabetic skills. Reading aloud to children is important, but unless combined with building skills in decoding the alphabet, it cannot lead by itself to literacy [15]. Reach Out and Read is the most widely known book distribution program in the pediatric space. It has been well-studied and research shows that the program improves language development [26], reading enjoyment [27], and parent-child bonding [28]. These are valuable outcomes for children and families; however, the available evidence does not demonstrate a substantial impact on children’s reading proficiency [24,29].

In contrast with distribution of books for reading aloud, there are currently no existing national programs that provide free toys for promoting early literacy. As a result, there remains an important gap in disseminating and implementing effective science-based interventions to help all our children learn to read. To our knowledge, this study is the first to look specifically at the availability and acceptability of alphabet toys and books in the HLE. Our findings demonstrate that families across socioeconomic strata value these tools, but that children from lower-SES households have significantly less access to them. We posit that this difference in availability is a modifiable contribution to the well-described equity gap in kindergarten-readiness.

There are several limitations to our study. We surveyed a convenience sample from one county in upstate New York. This limits generalizability, but we can think of no reason that the parents we surveyed would differ greatly in their views of home literacy tools from the broader general American public. We made a point of tabling at events happening in a variety of locations throughout the county and that had a variety of themes (e.g., some library events, a music festival, parent support group) to ensure a diverse group of respondents. An additional limitation was the use of zip code as a proxy for household income. We felt this approach was appropriate for this setting but recognize that the median income of a given geographic area does not necessarily reflect the income of the individual respondent. However, this approach yielded a high response: virtually everyone who approached the table completed the survey and answered all questions. We feel we would have had a much lower response if we had asked directly about income, eligibility for aid, or other such information. Additionally, although we decided a priori to examine those who had five or more items compared to those with zero items between the two established SES groups, when performing sensitivity analyses examining those who had 0–4 items compared to 5 or more, our findings remained significant in that those in the low SES group were less likely to have 5 + items in their homes.

A key strength of our study was that it was rooted in community engagement. Our survey was developed by and for community members living within Rochester, and our sampling occurred at diverse local events. The findings of the present study are a critical first step in identifying the needs of our local community and documenting the current perceptions of early literacy in this population.

These results support the need for and availability of joyful alphabet tools in low-income families. Intervention research is needed to establish the effectiveness of providing alphabet toys and books to children from lower SES backgrounds on equity gaps in kindergarten readiness, literacy, and long-term health outcomes. Our findings support the hypothesis that joyful letter-themed toys and books are acceptable to parents in general but that low-SES households are unlikely to have them. Closing the gap in early alphabet exposure could contribute to reducing inequities in school success and lifelong health.

## Supporting information

S1 Appendix Spreading the Joy of Literacy: A community-based survey of parents by parents. (PDF)
