# Supplementary material for: Spreading the Joy of Literacy: A community-based survey of parents by parents
Source: PLoS One. 2026 Nov 3;21(11):e0361893. doi: 10.1371/journal.pone.0361893 (PMC13730422; doi:10.1371/journal.pone.0361893)
Supplement: Supplementary file 1 [file pone.0361893.s001.pdf]

1. Do you think it is important that a child know most of the letters of the alphabet before Kindergarten?
    a. Yes
    b. No

2. How many of these items do you already have in your home? (Please look at the flyer with the pictures of the items)

    Number: _____________

3. Are there any children in your life that you would want to share these items with?
    a. No
    b. Yes
        i. If yes, who? (Circle all that apply)
            1. Children
            2. Grandchildren
            3. Nieces/nephews
            4. Cousins
            5. Neighbors
            6. All of the Above
            7. ____

4. Do the children in your life have their own library cards?
    a. Yes
    b. No
    c. __________________________________

5. Is there anything else you want to tell us about children learning to read or these learning tools? (Please provide your optional response in the space below)

6. What is your zip code? _____________________
