# Supplementary material for: Spreading the Joy of Literacy: A community-based survey of parents by parents
Source: PLoS One. 2026 Nov 3;21(11):e0361893. doi: 10.1371/journal.pone.0361893 (PMC13730422; doi:10.1371/journal.pone.0361893)
Supplement: Supplementary file 2 [file pone.0361893.s003.docx]

August 28, 2026

Rebuttal Letter for PONE-D-26-34608

Dear Dr. Clark, PLOS One Academic Editor,

We appreciate the feedback from all the reviewers and have used it to substantially improve this manuscript.

Responses to specific comments are listed below.

In general, we have clarified our use of the term Science of Reading and expanded our literature review to acknowledge the complexity of the process of learning to read. We have also addressed how alphabetic knowledge is relevant to different subgroups, including deaf children.

We believe we have now addressed the general and specific issues raised by the reviewers and await your response.

Sincerely,

Sarah Collins-McGowan, MD

Journal Requirements:

1. Please ensure that your manuscript meets PLOS ONE's style requirements, including those for file naming. The PLOS ONE style templates can be found at

[https://journals.plos.org/plosone/s/file?id=wjVg/PLOSOne_formatting_sample_main_body.pdf](https://urldefense.com/v3/__https:/journals.plos.org/plosone/s/file?id=wjVg*PLOSOne_formatting_sample_main_body.pdf__;Lw!!MkQhnT5yHH0MPvOc00c!U_a_Ag6OO38fwqJ4PZVxn0NHPDN8RUNnOGSliMCPb8rqHytAY-4sp4QUY2nOJS1YxWVTacH5EpfA6oVG2IvVi3scpnuhXXjrIIOJ$) and

[https://journals.plos.org/plosone/s/file?id=ba62/PLOSOne_formatting_sample_title_authors_affiliations.pdf](https://urldefense.com/v3/__https:/journals.plos.org/plosone/s/file?id=ba62*PLOSOne_formatting_sample_title_authors_affiliations.pdf__;Lw!!MkQhnT5yHH0MPvOc00c!U_a_Ag6OO38fwqJ4PZVxn0NHPDN8RUNnOGSliMCPb8rqHytAY-4sp4QUY2nOJS1YxWVTacH5EpfA6oVG2IvVi3scpnuhXVa3ZQZe$)

**AUTHOR RESPONSE:**

All file names have been adjusted to meet PLOS ONE’s style requirements.

2. In the ethics statement in the Methods, you have specified that verbal consent was obtained. Please provide additional details regarding how this consent was documented and witnessed, and state whether this was approved by the IRB

**AUTHOR RESPONSE:**

Verbal consent was obtained by the surveyor after the subject reviewed the information sheet. If verbal consent was obtained, the subject was given the survey to complete. If verbal consent was not obtained, no survey was administered. Consent was not documented separately, other than by completion of the survey.

An Ethical Considerations section was added to the Methods section clarifying the verbal consent process.

The IRB determined this study to be exempt. The protocol submitted to the IRB for review had the following language about consent:

“**Consent:** All potential subjects will receive an information sheet in English or Spanish describing the purpose of the study, the voluntary nature of the participation, and the rights of the subjects. See attached information sheet.

The study will not be collecting any Protected Health Information.

The study will ask people to provide their ZIP code. Otherwise there will be no potentially identifying data collected. ZIP code is being used as a proxy for socio-economic status.”

3. Thank you for stating the following in the Acknowledgments Section of your manuscript:

“This work could not have been done without the support of the JOLI Parent Advisory Board, the Wilson Community Pediatrics Fund, Rochester Area Community Foundation, Max and Marian Farash Foundation, Bright Start, Bloom Family Fund, Nancy Bloom and Alan Cohen, Beth Greenstein, Fairport Music Festival, Hipocampo Children’s Books, Roc the Future/PECAN, Monroe County Library System, WXXI PBS Kids, , Encompass, and University of Rochester Medicine Golisano Children’s”

We note that you have provided funding information that is not currently declared in your Funding Statement. However, funding information should not appear in the Acknowledgments section or other areas of your manuscript. We will only publish funding information present in the Funding Statement section of the online submission form.

Please remove any funding-related text from the manuscript and let us know how you would like to update your Funding Statement. Currently, your Funding Statement reads as follows:

“The project described in this publication was supported by the University of Rochester CTSA award number UL1 TR002001 from the National Center for Advancing Translational Sciences of the National Institutes of Health. The content is solely the responsibility of the authors and does not necessarily represent the official views of the National Institutes of Health.”

Please include your amended statements within your cover letter; we will change the online submission form on your behalf.

**AUTHOR RESPONSE:**

This study was entirely funded by the University of Rochester CTSA award as listed in our Funding Statement. The other funders listed in the Acknowledgements have funded previous work we have done. We have amended the Acknowledgements section to remove the names of funders. We have also removed funding related text from the manuscript.

4. Thank you for stating the following financial disclosure:

“The project described in this publication was supported by the University of Rochester CTSA award number UL1 TR002001 from the National Center for Advancing Translational Sciences of the National Institutes of Health. The content is solely the responsibility of the authors and does not necessarily represent the official views of the National Institutes of Health.”

Please state what role the funders took in the study.  If the funders had no role, please state: "The funders had no role in study design, data collection and analysis, decision to publish, or preparation of the manuscript."

If this statement is not correct you must amend it as needed.

Please include this amended Role of Funder statement in your cover letter; we will change the online submission form on your behalf.

**AUTHOR RESPONSE:**

The funders took no role in the study. The statement has been added to the cover letter and the manuscript.

5. Please note that your Data Availability Statement is currently missing the DOI/accession number of each dataset OR a direct link to access each database. If your manuscript is accepted for publication, you will be asked to provide these details on a very short timeline. We therefore suggest that you provide this information now, though we will not hold up the peer review process if you are unable.

**AUTHOR RESPONSE:**

This has been completed.

6. Please include your full ethics statement in the ‘Methods’ section of your manuscript file. In your statement, please include the full name of the IRB or ethics committee who approved or waived your study, as well as whether or not you obtained informed written or verbal consent. If consent was waived for your study, please include this information in your statement as well.

**AUTHOR RESPONSE:**

This has been completed. An Ethical Considerations section was added to the Methods section clarifying the verbal consent process.

7. We note that Figure 1 in your submission contain copyrighted images. All PLOS content is published under the Creative Commons Attribution License (CC BY 4.0), which means that the manuscript, images, and Supporting Information files will be freely available online, and any third party is permitted to access, download, copy, distribute, and use these materials in any way, even commercially, with proper attribution. For more information, see our copyright guidelines: [http://journals.plos.org/plosone/s/licenses-and-copyright](https://urldefense.com/v3/__http:/journals.plos.org/plosone/s/licenses-and-copyright__;!!MkQhnT5yHH0MPvOc00c!U_a_Ag6OO38fwqJ4PZVxn0NHPDN8RUNnOGSliMCPb8rqHytAY-4sp4QUY2nOJS1YxWVTacH5EpfA6oVG2IvVi3scpnuhXUfG4Dv9$).

We require you to either (1) present written permission from the copyright holder to publish these figures specifically under the CC BY 4.0 license, or (2) remove the figures from your submission:

1. You may seek permission from the original copyright holder of Figure(s) [#] to publish the content specifically under the CC BY 4.0 license.

We recommend that you contact the original copyright holder with the Content Permission Form ([http://journals.plos.org/plosone/s/file?id=7c09/content-permission-form.pdf](https://urldefense.com/v3/__http:/journals.plos.org/plosone/s/file?id=7c09*content-permission-form.pdf__;Lw!!MkQhnT5yHH0MPvOc00c!U_a_Ag6OO38fwqJ4PZVxn0NHPDN8RUNnOGSliMCPb8rqHytAY-4sp4QUY2nOJS1YxWVTacH5EpfA6oVG2IvVi3scpnuhXX4I_VPk$)) and the following text:

“I request permission for the open-access journal PLOS ONE to publish XXX under the Creative Commons Attribution License (CCAL) CC BY 4.0 ([http://creativecommons.org/licenses/by/4.0/](https://urldefense.com/v3/__http:/creativecommons.org/licenses/by/4.0/__;!!MkQhnT5yHH0MPvOc00c!U_a_Ag6OO38fwqJ4PZVxn0NHPDN8RUNnOGSliMCPb8rqHytAY-4sp4QUY2nOJS1YxWVTacH5EpfA6oVG2IvVi3scpnuhXUl5TPWw$)). Please be aware that this license allows unrestricted use and distribution, even commercially, by third parties. Please reply and provide explicit written permission to publish XXX under a CC BY license and complete the attached form.”

Please upload the completed Content Permission Form or other proof of granted permissions as an "Other" file with your submission.

In the figure caption of the copyrighted figure, please include the following text: “Reprinted from [ref] under a CC BY license, with permission from [name of publisher], original copyright [original copyright year].”

2. If you are unable to obtain permission from the original copyright holder to publish these figures under the CC BY 4.0 license or if the copyright holder’s requirements are incompatible with the CC BY 4.0 license, please either i) remove the figure or ii) supply a replacement figure that complies with the CC BY 4.0 license. Please check copyright information on all replacement figures and update the figure caption with source information. If applicable, please specify in the figure caption text when a figure is similar but not identical to the original image and is therefore for illustrative purposes only.

**AUTHOR RESPONSE:**

The images on Figure 1 have been replaced with images taken by the authors that comply with the CC BY 4.0 license.

8. If the reviewer comments include a recommendation to cite specific previously published works, please review and evaluate these publications to determine whether they are relevant and should be cited. There is no requirement to cite these works unless the editor has indicated otherwise.

**AUTHOR RESPONSE:**

Reviewer #1 recommended including work done by a number of other scholars and practitioners in the field of emergent literacy but did not recommend specific works. We have broadened our Background review of the literature to include several of the authors recommended by the reviewer, e.g. Shanahan. We specifically read the references recommended by the editor, as well as related articles, and have cited those: Easterbrooks, *et al*, Sampson *et al,* Andrews, Allen *et al.*

9. Please review your reference list to ensure that it is complete and correct. If you have cited papers that have been retracted, please include the rationale for doing so in the manuscript text, or remove these references and replace them with relevant current references. Any changes to the reference list should be mentioned in the rebuttal letter that accompanies your revised manuscript. If you need to cite a retracted article, indicate the article’s retracted status in the References list and also include a citation and full reference for the retraction notice.

**AUTHOR RESPONSE:**

We have reviewed our references, including additions made with this edit, and to the best of our knowledge, none of the cited papers have been retracted.

Additional Editor Comments:

Interesting split among reviewers. Reviewer one seems less familiar with CPAR than the second one. However, she is not wrong in that your two theories do hurt some groups of reader, deaf one especially due to phonological issues.

I would ask that you add more theory to your lit review and present both sides of the Simple View of Reading and the Science of Reading. try these articles for easy reviews
Easterbrooks, S. R., & Huston, S. G. (2008). The signed reading fluency of students who are deaf/hard of hearing. Journal of Deaf Studies and Deaf Education, 13(1), 37-54. for Simple View of Reading

Sampson, T., Becker, A., Campbell, E. E., Pyers, J., Hall, W. C., Sehyr, Z. S., ... & Caselli, N. (2026). Avoiding unintended consequences: science of reading policies may harm deaf children. Journal of Deaf Studies and Deaf Education, 31(3), 367-375.---this one for the Science of Reading issues

both of these reviews are with deaf readers who use more morphology but I still think that the letter/play would be important without these two theories. So maybe a braoder view of the many alternative pathways to reading (Andrews has that) and less on SVR and Science of Reading

I do not recommend any other changes to the article---it is a nice piece of CPAR

**AUTHOR RESPONSE:**

We are grateful for the editor’s comments. We expanded the Background section and clarified that our use of the term Science of Reading refers to the body of literature that exists on how children learn to read rather than a specific theory. We clarified that a common theme across subgroups of learners is that early exposure to the alphabet is important. We cited both papers recommended above by the editor. In addition we cited more recent related papers.


[Note: HTML markup is below. Please do not edit.]

Reviewers' comments:

Reviewer's Responses to Questions

**Comments to the Author**

1. Is the manuscript technically sound, and do the data support the conclusions?

The manuscript must describe a technically sound piece of scientific research with data that supports the conclusions. Experiments must have been conducted rigorously, with appropriate controls, replication, and sample sizes. The conclusions must be drawn appropriately based on the data presented.

Reviewer #1: No

Reviewer #2: Yes

2. Has the statistical analysis been performed appropriately and rigorously?

Reviewer #1: I Don't Know

Reviewer #2: Yes

3. Have the authors made all data underlying the findings in their manuscript fully available?

The [PLOS Data policy](https://urldefense.com/v3/__http:/track.editorialmanager.com/CL0/http:*2F*2Fwww.plosone.org*2Fstatic*2Fpolicies.action*23sharing/1/010f019fe995237e-68320ca1-4f67-4508-9e36-cdf7abb744a5-000000/v2KaXKDxvvnFYRvY6vZNooT_qOiVv_udrFFZ5lv5X8Q=258__;JSUlJSU!!MkQhnT5yHH0MPvOc00c!U_a_Ag6OO38fwqJ4PZVxn0NHPDN8RUNnOGSliMCPb8rqHytAY-4sp4QUY2nOJS1YxWVTacH5EpfA6oVG2IvVi3scpnuhXZIkn-_g$) requires authors to make all data underlying the findings described in their manuscript fully available without restriction, with rare exception (please refer to the Data Availability Statement in the manuscript PDF file). The data should be provided as part of the manuscript or its supporting information, or deposited to a public repository. For example, in addition to summary statistics, the data points behind means, medians and variance measures should be available. If there are restrictions on publicly sharing data—e.g. participant privacy or use of data from a third party—those must be specified.

Reviewer #1: Yes

Reviewer #2: Yes

4. Is the manuscript presented in an intelligible fashion and written in standard English?

PLOS ONE does not copyedit accepted manuscripts, so the language in submitted articles must be clear, correct, and unambiguous. Any typographical or grammatical errors should be corrected at revision, so please note any specific errors here.

Reviewer #1: Yes

Reviewer #2: Yes

5. Review Comments to the Author

Please use the space provided to explain your answers to the questions above. You may also include additional comments for the author, including concerns about dual publication, research ethics, or publication ethics. (Please upload your review as an attachment if it exceeds 20,000 characters)

Reviewer #1: Researchers neglect to review foundational theorists and practitioners who pioneered and developed the emergent literacy theory and practice. To name some: Marie Clay’s foundational work in Emergent Literacy in the 1960s; Jana Mason: challenged the “reading readiness” framework in the 1980s, advocating for more print exposure through simple storybooks on familiar themes; William Teale: emergent literacy theory; Elizabeth Sulzby: emergent literacy frameworks; Jerome Harste: added social interaction; Catherine Snow; Timothy Shanahan.

**AUTHOR RESPONSE:**

We appreciate the opportunity to respond to Reviewer #1’s comments and improve our manuscript. We re-examined the literature and added information on work done by Marie Clay in a reference by Timothy Shanahan. Shanahan was previously represented in the national reports that we had referenced. For the purpose of our study we did not feel an exhaustive review of emergent literacy theory and practice was necessary, but rather that it was important to demonstrate consistent findings of the importance of exposure to the alphabet.

While I buy the researchers’ thesis that poor families cannot afford and may not see the importance of letter-toys (because they are busy working, putting food on the table and paying the rent)—all children, no matter what the SES status see letters all around them in the environment, e.g, McDonalds, STOP sign, M and Ms candy, favorite foods, commercials on TV, Sesame Street, cartoons. What is needed are parents to talk to their children and show them how to make the link between meaning, printed letters, and sounds (if they are normally hearing). A better study would be to investigate parent attitudes toward early literacy activities. Sure, all parents can use letter magnets, letter toys, etc., but they simply need to look around them in the environment and point out letters to their young children from birth to five, prior to Kindergarten or first grade.

**AUTHOR RESPONSE:**

We agree that the best connections for children happen when parents, teachers, and other adults are actively helping them make the link between printed letters, meaning, and sounds. Children need repeated exposures to all letters as part of the learning process. While letters are all around in the environment, toys are meant for children and naturally grab their attention in a way that STOP signs do not. Having alphabet toys at home is a joyful way to increase exposure to letters for children, an additive effect to what is already in their environment. This study is community based participatory research where the study idea came from low-income mothers. The questions we asked were of specific interest to them.

Authors make no mention of the alphabet song, which every child learns to sing.

**AUTHOR RESPONSE:**

We are unsure how to respond to this comment. We do not discourage the alphabet song.

The “Science of Reading” and the “Simple View of Reading” have been debunked in the early reading literature, for hearing children as well as those with learning disabilities and special needs, including deaf children. Researchers make no note of that. Their reading theory section needs revamping. Other theories of early reading need to be acknowledged too to get a balanced picture.

In short, the paper lacks scholarship, and I cannot recommend acceptance.

**AUTHOR RESPONSE:**

We clarified in our Background section that our use of the term Science of Reading refers to the body of literature that exists on how children learn to read rather than a specific theory. We clarified that a common theme across subgroups of learners, including deaf children, is that early exposure to the alphabet is important. We have changed the language throughout to remove “oral” when that might confuse the issue. Part of the point of this paper is that existing programs do emphasize oral language while neglecting the alphabetic part of learning to read alphabetic languages, and so in a Both/And fashion, it would be good to work more on the alphabetic part.

Saying that the entire Science of Reading has been “debunked” is an extraordinary claim. It contradicts the existing evidence-based reviews like the National Reading Panel report and the National Early Literacy Panel report. If the reviewer would provide specific references for this point, we could address it more directly.

Reviewer #2: The work is well organized and easy to follow. This paper has the potential to positively impact literacy among children and youth. It addresses an important issue and provides valuable insights that could contribute to improving literacy skills and educational outcomes.

**AUTHOR RESPONSE:**

We appreciate Reviewer #2’s comment.

6. PLOS authors have the option to publish the peer review history of their article ([what does this mean?](https://urldefense.com/v3/__https:/track.editorialmanager.com/CL0/https:*2F*2Fjournals.plos.org*2Fplosone*2Fs*2Feditorial-and-peer-review-process*23loc-peer-review-history/1/010f019fe995237e-68320ca1-4f67-4508-9e36-cdf7abb744a5-000000/9Dwz4F5JNZmhQWgH_x7yoG2CIKEWsNfbNuw-nV5eRKQ=258__;JSUlJSUl!!MkQhnT5yHH0MPvOc00c!U_a_Ag6OO38fwqJ4PZVxn0NHPDN8RUNnOGSliMCPb8rqHytAY-4sp4QUY2nOJS1YxWVTacH5EpfA6oVG2IvVi3scpnuhXWLXMitx$)). If published, this will include your full peer review and any attached files.


If you choose “no”, your identity will remain anonymous but your review may still be made public.


**Do you want your identity to be public for this peer review?** For information about this choice, including consent withdrawal, please see our [Privacy Policy](https://urldefense.com/v3/__https:/track.editorialmanager.com/CL0/https:*2F*2Fwww.plos.org*2Fprivacy-policy/1/010f019fe995237e-68320ca1-4f67-4508-9e36-cdf7abb744a5-000000/IhjqcSiN0wLGIT1_cCoKsT6SJd123-QeOFFxnuHxh24=258__;JSUl!!MkQhnT5yHH0MPvOc00c!U_a_Ag6OO38fwqJ4PZVxn0NHPDN8RUNnOGSliMCPb8rqHytAY-4sp4QUY2nOJS1YxWVTacH5EpfA6oVG2IvVi3scpnuhXTmHTJ_I$).

Reviewer #1: No

Reviewer #2: No
